# Perceptions and acceptability of piloted *Taenia solium* control and elimination interventions in two endemic communities in eastern Zambia

**DOI:** 10.1111/tbed.13214

**Published:** 2019-06-24

**Authors:** Emma Clare Hobbs, Kabemba Evans Mwape, Andrew M. Phiri, Moses Mambwe, Richard Mambo, Séverine Thys, Gideon Zulu, Mwelwa Chembensofu, Chiara Trevisan, Inge Van Damme, Isaac Khozozo Phiri, Brecht Devleesschauwer, Jennifer Ketzis, Pierre Dorny, Arve Lee Willingham, Sarah Gabriël

**Affiliations:** ^1^ One Health Center for Zoonoses and Tropical Veterinary Medicine Ross University School of Veterinary Medicine Saint Kitts West Indies; ^2^ Institute of Tropical Medicine Antwerp Belgium; ^3^ Faculty of Veterinary Medicine Ghent University Merelbeke Belgium; ^4^ School of Veterinary Medicine University of Zambia Lusaka Zambia; ^5^ Ministry of Health Government of the Republic of Zambia Lusaka Zambia; ^6^ Sciensano Brussels Belgium

**Keywords:** communicable disease control, focus groups, parasites, public health, surveys and questionnaires, *Taenia solium*

## Abstract

Infections with *Taenia solium* cause significant public health and economic losses worldwide. Despite effective control tools, long‐term sustained control/elimination of the parasite has not been demonstrated to date. Success of intervention programs is dependent on their acceptability to local communities. Focus group discussions (FGDs) and questionnaires (QS) were conducted in two study communities in eastern Zambia to assess local perceptions and acceptance of two piloted intervention strategies: one targeting pigs only (‘control’ study arm), and one integrated human‐ and pig‐based (‘elimination’) strategy. QS (*n* = 227) captured data regarding participation in project activities, knowledge and perceptions of *T. solium* and of the interventional drugs used in the study. FGDs (*n* = 18) discussed perceived advantages and disadvantages of the interventions and of the project's delivery and value. QS data revealed 67% of respondents participated in at least one educational activity, and 80% correctly identified at least one disease targeted by the education. All elimination study arm respondents (*n* = 113) had taken the human treatment, and 98% intended to do so next time. Most (70%) indicated willingness to pay for future treatments (median 0.20 USD per dose). Of pig‐owning respondents, 11/12 (92%) had allowed their pigs to be treated/vaccinated and all intended to do so again next time. Four pig owners indicated willingness to pay 0.10–0.50 USD per dose of treatment or vaccine. FGD feedback revealed positive perceptions of interventions; people reported improved health in themselves and their pigs, and fewer cysticerci in pork. Latrine use, hand washing, meat inspection and proper cooking of pork had reportedly increased since the program's inception. Preliminary assessment indicates that the piloted intervention methods are generally acceptable to the communities. The reported willingness of many respondents to pay for the medications would contribute to the feasibility of long‐term, government‐led *T. solium* intervention programs in future.

## 1 INTRODUCTION

*Taenia solium* (the pork tapeworm) is a cestode parasite of humans and pigs, endemic through much of the developing world where free‐ranging pigs have access to human faeces. Humans are the definitive host harbouring the egg‐producing adult tapeworm (taeniosis, TS). Eggs are passed with the host's faeces, and when ingested by the intermediate host – usually scavenging pigs – lead to the development of cysticercosis (CC), in which larvae encyst within muscles and viscera. Eating infective cysticerci present in raw or undercooked pork products (porcine CC, PCC) leads to TS in humans and completes the life cycle; however, humans can also develop CC following accidental ingestion of eggs. Neurocysticercosis (NCC) occurs when the cysticerci develop in the intermediate host's brain and spinal cord and can cause a wide range of neurological conditions including seizures, chronic headache, stroke and hydrocephalus (Garcia, Nash, & Del Brutto, 2014). NCC is responsible for an estimated 5 million cases of epilepsy worldwide, making it the leading cause of preventable epilepsy in the developing world (Nash, Mahanty, & Garcia, 2013; Torgerson et al., 2015).

Although theoretically eradicable (CDC, 1993), high levels of active parasite transmission continue in many low‐income countries, where open defecation is practiced, access to medical and veterinary services is limited, health education is minimal, meat inspection is rudimentary or absent, and pigs are allowed to roam freely. Zambia is endemic for *T. solium*, with reported prevalence of taeniosis (6.3%–11.9%, by copro antigen‐ELISA), human CC (5.8%–14.5%, by serum Ag‐ELISA), NCC (57% of people with epilepsy) and PCC (up to 64% by Bayesian analysis) ranking among the highest in the world (Dorny et al., 2004; Mwape et al., 2012, 2013).

Effective tools for control of *T. solium* are available and include anthelmintics for humans and pigs, a pig vaccine, and non‐specific measures including health education, improvements to water and sanitation, and management of pigs. While numerous field studies have attempted to control *T. solium* using one or more tools with varying degrees of success in the short‐term (Ash et al., 2015; Lightowlers, 2010; Pondja et al., 2012; Sarti et al., 1997, 2000), only one recent large‐scale study in Peru has achieved significant interruption of parasite transmission to date (Garcia et al., 2016).

One of the key factors in achievement of sustained control or elimination is treatment coverage (Braae et al., 2016), which is largely reliant on the acceptability of intervention measures and the willingness of individuals and communities to participate. CYSTISTOP is a pilot intervention study that began in the highly endemic Eastern Province of Zambia in 2015, aiming to evaluate and compare an integrated short‐term *T. solium* elimination package versus a lower‐intensity control strategy. As part of this study, focus group discussions (FGDs) and questionnaires (QS) were conducted to assess the perceptions and acceptability of the piloted intervention strategies (human‐ and pig‐ oriented) in two study communities in the Eastern Province of Zambia.

## 2 MATERIALS AND METHODS

### 2.1 Study area

The study was conducted in the Nyembe and Chimvira communities, in the adjoining Katete and Sinda districts of the Eastern Province of Zambia, respectively. The Nyembe community contains approximately 1,200 people in 227 households, nominally divided into eight villages, although boundaries are loosely defined. The Chimvira community is divided into eleven villages and contains approximately 1,400 people in 325 households. The study areas were selected based on previous epidemiological studies in the region, occurrence of risk factors, accessibility throughout the year and community willingness to participate.

Over half of the nation's estimated 1.25 million pigs are reared in the Eastern province, largely under small‐scale free‐ranging conditions (ZDA, 2011). Pig slaughter in rural areas occurs informally with little to no meat inspection, although tongue palpation for identification of PCC at point of sale is increasingly common thanks to ongoing education campaigns. A preliminary costing study in the region determined that 67% of pig sales include tongue palpation, and that 95% of tongue‐positive pigs cannot be sold, representing substantial economic losses for subsistence pig farmers (Hobbs, Mwape, Devleesschauwer, et al., 2018).

### 2.2 Study design and delivery

CYSTISTOP is a community‐based interventional pilot project that began in Zambia in 2015. While CYSTISTOP will continue until late 2020, the study at hand relates specifically to data collected via FGDs and QS conducted in the elimination (November 2016) and control (March 2017) study arms.

#### 2.2.1 Interventions

In the ‘elimination’ study arm (Nyembe community), integrated intervention strategies consisted of human mass drug administration with an anthelmintic (praziquantel, PZQ at 10 mg/kg orally for most participants, or niclosamide at 2 g orally, for individuals who tested serologically positive for CC at baseline), plus deworming (oxfendazole, OFZ at 30 mg/kg orally) and vaccination (TSOL‐18 1 ml intramuscularly) of pigs. These interventions were conducted at four‐monthly intervals. In the ‘control’ study arm (Chimvira community), deworming of pigs with OFZ was conducted at baseline and then annually.

Health education was also conducted concurrently with interventional activities in both study arms. Village‐based educational sessions were held at the beginning of each intervention visit. Sessions were delivered in Chewa (the local language) by one of the project's principal investigators and utilized educational aids including a large canvas poster of the *T. solium* life cycle, A4‐sized laminated photographs of the parasite's different developmental stages, life‐sized plasticine models of human stool containing tapeworm segments and a five‐metre long ribbon to represent the actual size and length of the adult tapeworm. Sessions were approximately 30 min in duration and encouraged input and questions from the audience. Other educational activities included simple A4‐sized informational flyers which were distributed and explained to individual households, larger educational posters that were permanently displayed at the rural health centres and educational workshops for primary school students using the computer‐based educational program ‘The Vicious Worm’ (Hobbs, Mwape, Van Damme, et al., 2018; Hobbs et al. 2019).

#### 2.2.2 Baseline sampling survey

A baseline survey collected human blood and stool samples, and pig blood samples and carcass dissections for determination of human CC and TS, and PCC prevalence, respectively. Blood samples from eligible humans and pigs were collected by trained medical and veterinary members of the project team, respectively, according to standard hygienic venipuncture practices. Human stool samples were donated by willing individuals following distribution of stool pots by project team members.

#### 2.2.3 Data collection

Perceptions of the project's delivery and value were qualitatively assessed via feedback from FGDs. Acceptability of the piloted interventions was assessed using quantitative measures of intervention uptake and project participation, and economical valuation of interventional treatments obtained via QS. Qualitative feedback of project acceptability was also obtained via FGDs.

**Figure 1:**
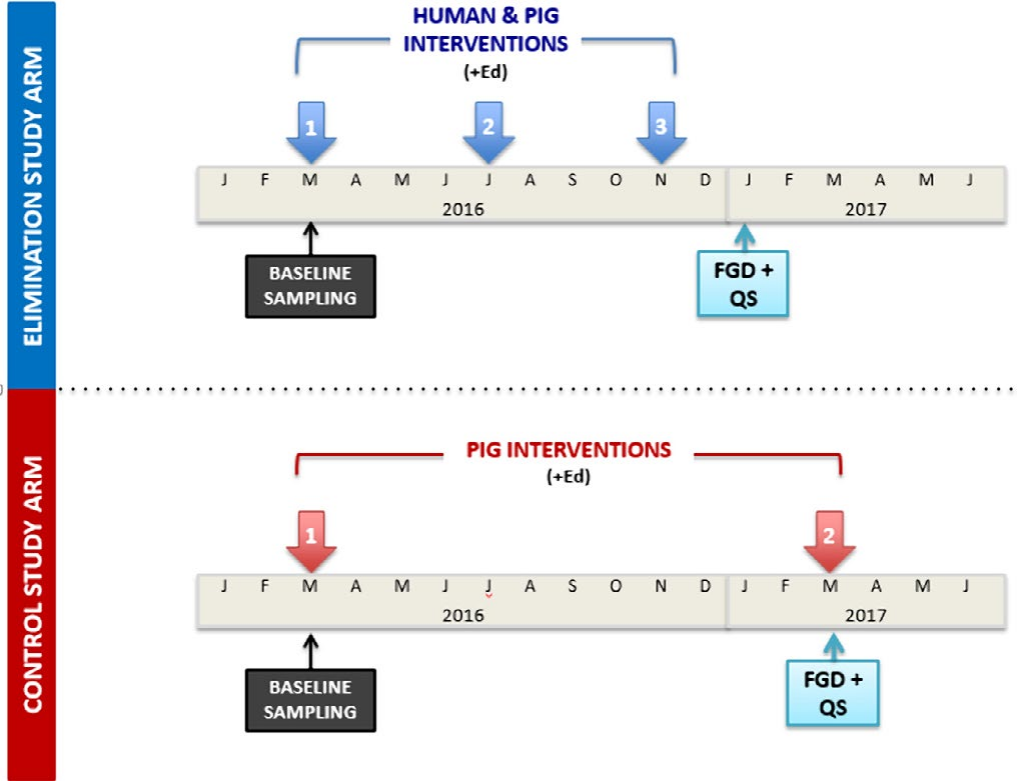
Schematic representation of the CYSTISTOP project activities during the period described in this study. Ed: human health education; FGDs: focus group discussions; QS: questionnaires

Data collection in the elimination study arm took place in November 2016, after the third intervention round, and in the control study arm in March 2017, after the second intervention round. The baseline sampling survey had taken place in March 2016, immediately prior to the start of interventions (Figure 1).

#### 2.2.4 Questionnaires (QS)

The perception QS used in the study villages (Files S1 and S2) were developed by study team members in conjunction with the team's consultant anthropologist. The final QS were written in English upon request from QS survey teams during the pre‐testing, but specific terminology and translations into Chewa were discussed and agreed upon during training sessions prior to implementation in the field. The QS were entered into the electronic mobile data collection program Magpi 2.0 (https://home.magpi.com/) and uploaded onto smartphones for mobile data collection.

Fifty per cent of consenting households in each of the two intervention study arms were randomly selected for QS administration. QS were administered door‐to‐door in Chewa by trained program staff, ideally with the household head or, if they were unavailable, with another adult household member. Respondents were asked about their knowledge of *T. solium* TS/CC, their perceptions of the interventions conducted in their villages, and their opinions regarding the delivery and value of the project.

#### 2.2.5 Focus group discussions (FGDs)

Discussion themes (File S3) were developed by study team members in collaboration with the consulting anthropologist. Themes were designed to determine participants’ perceptions of the acceptability, importance and value of each of the intervention activities conducted in their villages and to elicit suggestions for what could be improved in future programs.

All FGDs were conducted in Chewa by the same trained facilitator from the University of Zambia, who was independent of the CYSTISTOP team, and held in neutral locations (rural health centre, primary school) in an effort to reduce sponsor bias on the FGD participants. Equal numbers of adult male and female FGDs were conducted, and discussions lasted approximately 1 hr in duration. Some of the smaller villages in each of the study arms were combined (Table 1), and group sizes ranged from five to ten participants depending on the size of the village/s that were represented. Adult participants aged 18 years or older were purposively selected from the villages following recommendations from local community health workers about the suitability of their characters (willingness to speak their minds and engage in the discussions) and being representative of different areas of the community, as well as their individual availability and willingness to participate. All sessions were videotaped to facilitate data transcription, following oral agreement from all members in each of the groups. All participants were part of the CYSTISTOP study population and had signed an informed consent at the start of the study. The number of FGDs was sufficient to reach data saturation, in which no additional themes were raised.

**Table 1:** Village and gender groupings of FGDs conducted in the two study arms

Name/s of villages represented in focus group discussion	Number of focus group discussions	No. of attendees – Male group	No. of attendees – Female group	No. of attendees – total for village/s	Total population at baseline
**Elimination study arm**	8	25	34	59	1,027
Chikhutu	2	8	10	18	289
Kayela‐Zonde^a^	2	6	9	15	224
Mtalalika	2	5	6	11	272
Safari	2	6	9	15	242
**Control study arm**	10	41	44	85	1,347
Azele‐Chimphafa‐Chibweza‐Kamlawe^a^	2	6	9	15	181
Chimvira	2	8	8	16	367
Kabonga	2	8	8	16	236
Kamkulekule‐Chikuse‐Kamvumvuli^a^	2	9	9	18	311
Mkambi‐Mfunanji^a^	2	10	10	20	252
**Totals**	18	66	78	144	2,374

a Villages were combined.

### 2.3 Data processing and analysis

Questionnaire data were uploaded to the Magpi server and exported to Microsoft Excel for descriptive analysis. A conversion rate of 1 Zambian kwacha to 0.10 USD (United States of America Dollars) was used where applicable. Recorded footage of FGD sessions was transcribed into English, and transcripts uploaded into the Weft QDA qualitative data analysis program (v1.0.1, 2006). The major themes were separately identified through coding following an inductive approach (Thomas, 2006).

### 2.4 Ethical statement

The protocol and procedures for the ongoing CYSTISTOP project were reviewed and approved by the University of Zambia Biomedical Research Ethics Committee (004‐09‐15, covering both human and animal ethical clearance) and the Ethical Committee of the University of Antwerp, Belgium (B300201628043, EC UZA16/8/73) as well as the Institutional Review Board of Ross University School of Veterinary Medicine, St Kitts. The study was introduced and explained to all project participants, both in village group sessions and within individual households, during each field visit. Written informed voluntary consent was given by all participants in the CYSTISTOP project, and participation in QS and FGD activities was entirely voluntary. No incentive was given for participation in the QS or FGDs, although light refreshments were provided after FGDs. During transcription of FGD footage, participant names were omitted to protect anonymity.

## 3 RESULTS

### 3.1 Questionnaire (QS)

The QS were completed by 227 individuals: 113 from the elimination study arm (representing 49.8% of households) and 114 from the control study arm (representing 35.1% of households). Of the total respondents, 145 (64%) were female. Pigs were owned by 12 households, of which 9 (75%) were in the elimination study arm.

Participants’ knowledge of *T. solium* was variable: 68% (155/227) of respondents correctly stated that the educational messages were targeting CC (‘masese’ in Chewa) and 49% (112/227) mentioned epilepsy. Only 30% (69/227) specifically mentioned TS, while 33% (74/227) mentioned worms in general. Overall, 88% (200/227) of surveyed respondents perceived *T. solium* as a serious health concern for people.

While only 5% (12/227) of the total respondents owned pigs at the time of the QS, 79% (180/227) of total respondents correctly stated that the pig treatments used in the study were targeting PCC, with 31% (70/227) also correctly indicating that the treatments were also effective against other worms. Twenty respondents (9%), nearly all of whom were from the control villages, incorrectly stated that the treatments were for African swine fever (ASF).

In the elimination study arm, respondents were also asked if they knew what the human interventions with PZQ were used to treat. Of the 113 respondents, 86 (76%) correctly chose ‘worms’, and one person mentioned schistosomiasis. (While PZQ is used to treat schistosomiasis, the required dosage is four times the 10 mg/kg used in this study for treatment of TS.)

#### 3.1.1 Educational activities (both study arms)

**Figure 2:**
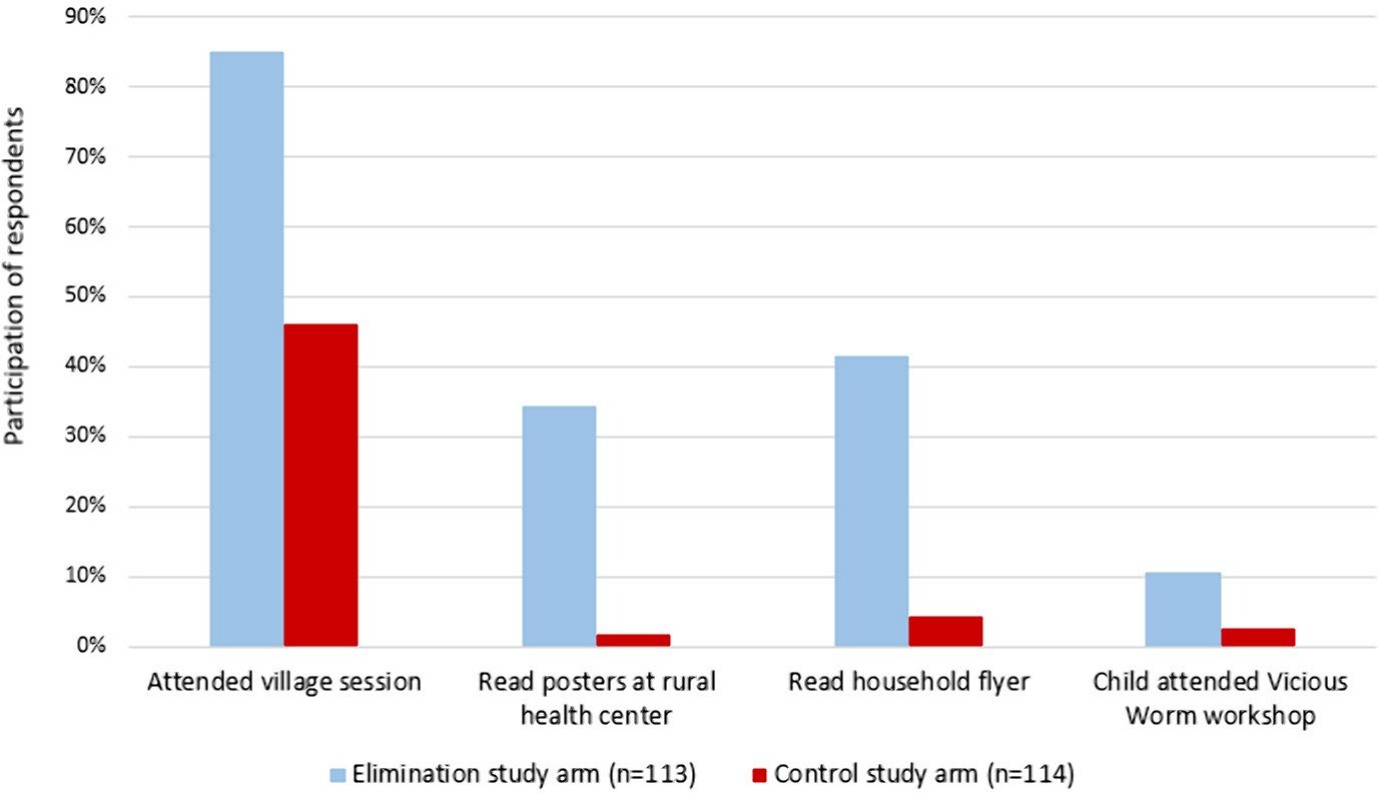
Participation of questionnaire respondents in CYSTISTOP educational activities in the two intervention study arms

Two‐thirds of QS respondents had reportedly participated in at least one of the four CYSTISTOP educational activity types. Overall participation was higher in the elimination study arm than in the control area, with 89% (101/113) of respondents from elimination villages participating in at least one educational activity, compared to 46% (52/114) in the control study arm in which educational activities were delivered much less frequently. The village educational sensitization sessions were the best‐attended activity in both study areas (85%, 96/113 in elimination arm, 46%, 52/114 in control arm) (Figure 2). Village sessions were also perceived to be the best of the educational activities (voted by 87% of respondents for that question, 45/52.)

In the control study arm, 45 of 52 respondents (87%) stated that they had discussed the content of the educational sessions with others. Most of the discussions (28/45, 62%) were with family members, and the remainder (17/45, 33%) were with friends. (No response data were given in the elimination study arm.)

There were 74 individuals (33% of 227 total respondents) who did not reportedly participate in any educational activities. The most common reason for non‐participation was having other priorities at the time of the visits, including working in the fields, caring for a baby or ill relative or being away from the village. Three individuals were reportedly unable to participate because they did not have permission from their household head. Three others stated a lack of interest as the reason for their non‐attendance.

#### 3.1.2 Human interventions (elimination arm only)

All 113 respondents from the elimination study arm had taken the PZQ treatment at the most recent intervention, and 111 (98%) indicated that they would do so again at the next intervention. Almost two‐thirds of respondents (80/113, 29%) reported that they experienced no side effects following treatment. Side effects were reportedly experienced by 33 individuals (29% of the 113 respondents), the most common being dizziness (17/113, 15%), followed by headache (10/113, 9%), and then diarrhoea (5/113, 4%), all of which were described as mild and self‐limiting. Of the 33 people who reportedly experienced side effects, 23 (70%) reported a single complaint, while nine individuals experienced two and one individual reportedly experienced three complaints (headaches, dizziness and joint pain). The two people who did not intend to take the PZQ at the next intervention had reportedly experienced side effects (one had diarrhoea, and the other dizziness and headaches) and both listed this as the reason for their intention to refuse subsequent treatments.

Over two‐thirds (99/113, 70%) of total surveyed respondents indicated a willingness to pay for PZQ treatments in future, and the median suggested price was 0.20 USD per dose (range 0.10–5.00 USD per dose).

#### 3.1.3 Pig interventions

Of the 12 pig‐keeping respondents, 11 (92%) had allowed their pigs to be included in the most recent round of interventions, and all 12 indicated that they would do so at the next round. One pig in the elimination study arm reportedly demonstrated mild, self‐limiting anorexia after an intervention. No other side effects were reported by the other pig owners.

Four pig owners (33%), all of whom were from the elimination study arm, indicated willingness to pay for the medications in future. Median suggested prices for one treatment with OFZ was 0.43 USD and for one dose of TSOL‐18 vaccine was 0.38 USD, with prices for both ranging from 0.10 to 0.50 USD.

### 3.2 Focus group discussions (FGDs)

A total of 144 people participated in the 18 FGDs: 59 from the elimination study arm and 85 from the control study arm. Total male and female proportions were 46% and 54%, respectively. The results highlight the different themes that arose during the analysis, with anonymous quotes included for illustration of relevant or representative opinions. Subheadings indicate whether topics were applicable to one or both study arms. For topics that were applicable to both study arms, the same themes were observed at similar frequencies across all villages and study arms.

#### 3.2.1 People's perceptions of human treatments (elimination study arm only)

Many people stated that they had noticed better overall health in themselves or family members since taking the treatments, with specific examples including increases in weight and appetite, less bloating and improved bowel functions. Additional reported benefits included a reduction in neurological conditions including seizures, epilepsy, dizziness and headaches:

When you drink that medication, I have experienced that one doesn't have frequent headaches. From the last time I drank the medicine, I have not had headaches I used to experience on monthly or weekly basis. (Focus group/Women/Kayela‐Zonde)

Some respondents reportedly observed tapeworms being expelled after the treatments:

After I chewed [the tablets], the following morning after I went to the bush, I defecated a long worm with different segments. From that time until now I have never experienced any problem, whatsoever. (Focus group/Men/Mtalalika)

Negative comments were limited to reports of unpleasant side effects from the medication, including headache, dizziness, abdominal pain, vomiting and constipation. These were all described to be mild and self‐limiting.

Speaking of my own thoughts: after taking those medications, I felt bad. I felt like I had contracted an illness because my head really pounded but after two days the headache subsided. (Focus group/Women/Chikhutu)

#### 3.2.2 People's perceptions of human blood and stool sampling for diagnosis (both study arms)

Several respondents from both study arms expressed initial distrust in the program's requests for stool and blood sampling, which led to several people refusing to submit samples in the first sampling round. The most commonly reported reasons for distrust were fears that the program was satanic, or was collecting blood to be sold for profit. Some people also reported feeling initially apprehensive due to a lack of understanding about the process, wondering for example how much blood would be collected, and what it would be used for. One person also indicated that there was some fear surrounding what a positive test result might mean:

…Some people could have challenges understanding exactly what it meant when they were found with the infection. They were wondering whether they were about to start convulsing. (Focus group/Women/Kamkulekule‐Chikuse‐Kamvumvuli/Control study arm)

Groups acknowledged the high numbers of people experiencing seizures in their villages, and some indicated that diagnosing epilepsy as a result of parasitic infection appeared to dispel some traditionally held beliefs about it being caused by God, witchcraft or cursing, although this was not consistently reported. Some respondents indicated that people with epilepsy were now more likely to seek and receive treatment from the rural health centres as opposed to visiting traditional healers as they did in the past.

About the issue of epilepsy, we now know that there is something affecting the brain. Previously and going by our fore fathers, we thought it was all about witchcraft. We thus used to visit witch doctors, which was not helping at all. When a person died we concluded that the one responsible was very powerful (more so than the witch doctor). (Focus group/Women/Azele‐Chimphafa‐Chibweza‐Kamlawe/Control study arm)

During the FGDs, there was consensus that the initial fears and distrust in the program had disappeared over time. People cited developing trust in the program team over time, improved knowledge after attending educational sessions, understanding the rationale behind the sampling surveys and the tangible health benefits in the human and pig populations, as reasons for this. Many respondents provided personal testimonials to their changing perceptions and behaviours:

“How can my husband let our child submit blood?” I said in my heart, “Those are Satanists who have come here!” They collected blood from that child, but I was quiet. My husband proceeded to have his taken. I was convinced that he had handed over my child to Satanism. But now, even I am prepared to give a blood sample. (Focus group/Women/Mtalalika/Elimination study arm)

People who had submitted samples were appreciative of receiving their test results and valued knowing the health status of themselves and their family members.

Personally I was very happy to test negative from the samples that I submitted. I would be more than willing to be tested again. Who knows I may be infected this year, 2017. (Focus group/Men/Kamkulekule‐Chikuse‐Kamvumvuli/Control study arm)

Some respondents reported dissatisfaction that only two household members were serologically tested for CC at baseline and wondered why the whole family was not tested.

I was…thinking that they should have taken blood samples from everyone at the household so that everyone [knows] their status. But upon asking them when they came during the meeting, they explained that it's costly to test all the collected blood samples from the village. So that was one of the reasons why only two were selected from each household, a child and an adult. If the finances could allow, all in the village could have been sampled. (Focus group/Women/Mtalalika/Elimination study arm)

It was mentioned that some individuals who had initially refused to submit samples later regretted their actions, and there were several testimonials from people who had initially refused but were later inspired to participate in subsequent sampling surveys.

#### 3.2.3 People's perceptions of pig interventions (oral medication in both study arms, additional vaccination in elimination study arm only) and blood sampling (both study arms)

Opinions of the pig interventions were mixed, with several FGD participants stating that it was difficult to report on the efficacy of the treatments and/or vaccinations given the large decrease in pig populations in the study areas.

…Those pigs died…by the time you were coming to treat them they were already diseased. At Chimvira village, for instance, we had about 800‐plus pigs. About 700 of those died meaning that they had already acquired the disease. We are wondering whether the medication was of any benefit or not. (Focus group / Women / Azele‐Chimphafa‐Chibweza‐Kamlawe, Control study arm)

There are no pigs in the village. So, it is very difficult to ascertain whether the vaccinations are working or not because pigs died from ASF. Even the treated ones died on their own. It is very difficult to testify to the efficacy of the vaccinations, whether they would work or not. For sure! (Focus group/Men/Mtalalika/Elimination study arm)

In both study areas, people indicated that there were some concerns that the pig interventions and/or blood sampling activities were responsible for the pig deaths in the area. Most respondents went on to say that it was generally accepted that the pigs were dying due to ASF, often after learning that pigs outside the treatment areas were similarly affected.

M3: When these [program] people came, we had a challenge with ASF. They were collecting blood from some pigs, putting ear tags, and weighing them. When they left, many of those pigs died. People then started wondering as to why many pigs died after the exercise.

M1: …After blood was collected, the pigs died. But then, I cannot say they died because of the treatment. Here we have a problem of ASF. (Focus group/Men/Azele‐Chimphafa‐Chibweza‐Kamlawe/Control study arm)

Some people have been thinking that the pigs died of the treatment. Taking a close look, we realize that even pigs from far areas including our neighboring Mozambique have been dying. I feel you meant well but the treatment coincided with ASF. (Focus group/Women/Chimvira/Control study arm)

There did appear to be some residual concerns about this issue in the study areas, seemingly associated more pig vaccinations than oral treatments. This reportedly led to some pig owners refusing to let their pigs be vaccinated:

W9: The time vaccinations were taking place, the pigs were unwell. After vaccinations, many of them died.

W5: Many are complaining saying those injections that were given resulted in my pig dying.

W7: Others have refused [vaccinating] this time around. (Focus group/Women/Kayela‐Zonde/Elimination study arm)

Respondents in both study arms stated that of the surviving pigs, those that had been treated with/without vaccinations appeared in good health:

Those [treated pigs] with ear tags look different from the rest because they are very healthy. One would wish to have such pigs. (Focus group/Men/Mkambi‐Mfunanji/Control study arm)

Several people also indicated that they had noticed a decrease in cysticerci in pork meat since the beginning of the program:

…Hearing from those who slaughter pigs, they say they are encountering very few pigs with cysts, and when they do the cysts appear destroyed. We have testimony that the medication really cures the disease. (Focus group/Women/Mtalalika/Elimination study arm)

#### 3.2.4 Perceptions of educational sessions (both study arms)

In both study arms, participants gave positive responses about the educational sessions, often citing the educator's use of photographs and the long ribbon to represent the adult tapeworm, as being effective methods. Many individuals were able to correctly describe the life cycle of the parasite, and how it causes disease in people and in pigs:

We were taught about ‘masese’: going into the bush and defecating there allows pigs to come and eat the excreta. When those pigs have eaten the excreta and if you have the tapeworm the pigs can contract the disease. When such a pig is eaten, man gets back the disease. Therefore, when preparing this pork it must be cooked, boiled for a long time before eating. (Focus group/Women/Kayela‐Zonde/Elimination study arm)

Even in instances where a person had confused parts of the life cycle (for example, incorrectly stating that epilepsy is caused by eating infected pork), they were still able to correctly state the key messages for preventing disease transmission, such as importance of hand washing and proper cooking of pork, among others. Furthermore, most participants stated they had in fact implemented behavioural change as a direct result in the educational messages, and many also reportedly encouraged their children, families and others to do so.

#### 3.2.5 Reported behavioural changes arising as a result of the program (both study arms)

In all groups, respondents reported implementing behavioural changes in order to reduce ‘risky’ behaviours that promote disease transmission. There was consensus across both study areas that since the program's commencement there was much increased usage of latrines, including for the disposal of babies’ and children's excrement, and more regular handwashing using soap or ash, including after using the toilet, before preparing food and breastfeeding babies. Behavioural changes relating to food hygiene included increased use of dish racks to elevate cooking equipment from the ground; proper cooking of pork instead of only roasting the outside; and increased meat inspection by local environmental health workers, leading to infected pork or pig carcasses being buried or destroyed instead of sold or eaten. Many people also recognized the crossover benefits of these practices in preventing other – particularly diarrhoeal – diseases.

Coming to the issue of pork, in the past we used to eat infected meat without realizing the risks involved. Even after noticing that it was infected with ‘masese’, we could still go ahead and eat. The coming of this program has seen an improvement in that area. People now ensure that slaughtered animals are inspected first before consumption. If it is found to be diseased, such meat is destroyed. This has been helpful in the prevention of diseases such as seizures. (Focus group/Men/Mkambi‐Mfunanji/Control study arm)

Many groups discussed the social nature of disease spread and recognized that infections do not just affect individuals but also households and communities. While men were often cited as being most likely to continue consuming undercooked pork – particularly in conjunction with beer drinking – understanding the parasite's life cycle made people realize that even those who do not consume pork are at risk of acquiring CC (‘masese’).

It may happen that, without washing hands, one would greet a colleague through a handshake. In that case a colleague would have got ‘masese’. It may also happen that you touch food and in that way ‘masese’ would be transmitted to others. (Focus group/Men/Azele‐Chimphafa‐Chibweza‐Kamlawe/Control study arm)

I also learned that even those that do not eat pork can be infected. Water can wash away the eggs and deposit them into gardens or fields. Fruits such as mango can fall on the ground and once eaten without washing, one can get infected. (Focus group/Women/Chimvira/Control study arm)

People also stated having educated pork traders on the dangers of infected pork:

…If we have seen him selling pork with cysts and we have encountered him, we can challenge and advise him that, “You, this meat is not good for humans as it brings diseases to them. …This meat is not supposed to be sold here. It needs to be discarded so that we can be helped in our area. (Focus group/Men/Chikhutu/Elimination study arm)

Significant increases in both construction and usage of toilets in study areas were repeatedly identified by FGD participants as being of particular benefit. Having a latrine was associated with comfort and convenience, as well as being a source of dignity and respect, particularly when visitors or in‐laws come to the house:

A toilet is indeed very important because even as you receive visitors, you feel free. Usually a visitor is fed and after that they may want to use the toilet. It may be very shameful to direct your visitor to the bush. If you have a toilet then you will be able to save yourself from the shame. (Focus group/Men/Kabonga/Control study arm)

Many groups discussed the absence of latrines in or near the planting fields, which are often located quite some distance from the villages. While open defecation was normally practised in these areas in the past, most groups were reportedly now using the ‘cat method’, in which a hole is dug and excreta buried to prevent scavenging pigs from accessing it. The value of preventing scavenging pigs from accessing human stool was discussed and acknowledged by many participants; however, confining pigs was largely prevented in practice by the lack of alternative feed sources they would require.

…[It is] very challenging to confine pigs here because of lack of adequate food. Confining pigs calls for adequate provision of food on a daily basis. If we cannot fend for ourselves, how can [we] manage with pigs? So they said they were not there to stop us from keeping pigs but find alternative means and ways of preventing diseases in both pigs and humans. (Focus group / Men / Mkambi‐Mfunanji / Control study arm)

Repeated educational sessions were reportedly effective at dispelling people's initial fears and distrust in the program, especially with regards to Satanism. The enthusiasm with which many people participated in interventions and sampling was cited as proof of their confidence in and appreciation for the program:

Moderator: What happens when the vehicles come into your villages?

W6: The kids shout that there go the ‘masese’ people!

W1: Kids now drag their parents saying, “Let us go and drink the medication! (Focus group/Women/Kayela‐Zonde/Elimination study arm)

The success of the CYSTISTOP program in achieving behavioural change was highlighted by comparisons with a previous program's unsuccessful attempts to promote latrine use, due largely to the absence of accompanying education:

In the past, we never used to appreciate the importance of toilets and we thought that they were forcing us for no reason. Now, we have come to realize and understand how useful and important a toilet is. (Focus group / Men / Mkambi‐Mfunanji / Control study arm)

Several respondents stated that living in an intervention area was a privilege, and that they felt a responsibility to educate neighbours and friends from other areas so that they could also benefit from the program. This was partly altruistic but also due to recognition that people from other areas may act as sources of infection.

…We were taught that we need to wash our hands after using the toilet. Now some people may go without doing that; infecting others. That is why I am saying that there is need for the entire area to be informed adequately about these issues. (Focus group/Women/Kamkulekule‐Chikuse‐Kamvumvuli/Control study arm)

…You saving me may in turn make me save many other lives as well. Because of your advice that you will have given me, I will enlighten other people, too. (Focus group / Men / Chikhutu / Elimination study arm)

#### 3.2.6 Observations on gender and hierarchy (both study arms)

All groups were consistent in their interpretations of gender roles in the villages. Men are responsible for building latrines, while women – and sometimes children – are responsible for latrine maintenance (such as refilling the hand washing stations, adding ash to the pit latrines to reduce odours and replacing the latrine cover to prevent flies.) Women also prepare the food and care for children and babies.

Many groups of both sexes mentioned the tendency of men to drink beer, sometimes to such an extent that they squander the limited income of the household, and may also neglect their duties at home, such as failing to build latrines or other facilities.

…Some people…still do not have toilets, dish racks, bathing shelter and other facilities. That is why women complain about some of these men. They have got too much into the issue of beer drinking. They are ever drunk; no strength to do anything. (Focus group/Men/Kamkulekule‐Chikuse‐Kamvumvuli/Control study arm)

Another negative outcome raised in the discussions is the tendency of men to eat roasted (undercooked) pork while drinking, therefore increasing their risk of acquiring TS.

The position of household head can be held by both men and women, and whoever holds that role is ultimately responsible for making decisions for the rest of the household members. Village leaders, on the other hand, are almost always male (‘headmen’). Headmen can implement rules of conduct for all individuals in their villages, and can be appealed to discipline people for bad behaviour, or to intervene in household or neighbourly disputes. All headmen of villages within a region report to the area chief. The current area chiefs of the study districts are both male, however, there have been female chiefs in the past. The area chief is ultimately responsible for the oversight of all villages and inhabitants within their area.

During the design phase of the CYSTISTOP project, several areas within the Eastern Province were shortlisted for possible enrolment in the study. Meetings with area chiefs and village headmen were conducted, and their enthusiasm for and willingness to participate in the project were among the key selection criteria for enrolment in the project. The area chiefs and village headmen in the three study areas chosen for the CYSTISTOP project have been active supporters of the project since it commenced in 2015. CYSTISTOP team members regularly meet with the area chiefs to discuss the project's progress. CYSTISTOP meetings are conducted with all of the village headmen within each study area at the beginning of each field visit, in order to re‐affirm the project's goals, convey results and progress of the project since the previous visit, and to discuss any questions or challenges that may have arisen, so as to develop and agree on local solutions. All of the headmen play active roles within their villages in varying ways; examples include vocalizing their support of the project during the village‐based educational sessions, allowing CYSTISTOP teams to set up treatment stations from their households, and personally following up with individual villagers to encourage their participation in project activities.

#### 3.2.7 People's recommendations for the program in future (both study arms)

All groups were united in requesting that the program continue, both for the health benefits of the human and pig treatments, and for the ongoing education of people who may have been absent, or who might take longer to comprehend the key messages than others.

What we may implore is that you the owners of the program need to continue to educate people because some take time to assimilate and understand. You need to be repeating such awareness programs so that they do not forget. (Focus group/Men/Chikhutu/Elimination study arm)

Many people also requested that the program expands to neighbouring areas, both for the benefit of others as well as to protect themselves. One person suggested that the program could broadcast educational sessions on the local radio station, as an effective way of spreading the message to a wider audience. Other recommendations included construction of additional boreholes, and making laws that require all households to have toilets, although it was acknowledged that these could be implemented locally, by village headmen, and not the program. The need for development of a vaccine for ASF was also discussed.

## 4 DISCUSSION

The results of this study indicate that most of the piloted interventions (human and pig treatments and sampling) and educational sessions in the study areas are appreciated and valued by the local communities. This can be inferred from direct testimonials from FGD participants, as well as by the high participation rates in program activities and the stated willingness of many individuals to pay for the treatments and/or vaccine in future. Non‐participation in program activities was reported to be largely due to other commitments rather than a lack of interest. Educational activities were highly acceptable to the communities, particularly in the elimination study arm due to increased frequency, and respondents perceived them to be a valuable component of the project. Over 87% of QS respondents had reportedly discussed the program's educational content with family members and friends, which is likely to have enhanced knowledge uptake and transfer in these communities.

Perceived benefits of the intervention activities included purportedly healthier people and pigs, fewer cysticerci in pig carcasses during slaughter, and higher awareness and understanding of the parasite, which reportedly led to behavioural changes including greater latrine use, more handwashing, and improved food hygiene. Although the exact details of the *T. solium* life cycle were not completely understood by all study participants – as indeed is often seen even among veterinary and medical professionals (Devleesschauwer, Smit, Dorny, van der Giessen, & Gabriel, 2015; Ertel, Braae, Ngowi, & Johansen, 2017) – the key messages for disease prevention were evidently well retained. While arising from a *T. solium*‐specific program, these behavioural changes are also likely to decrease transmission of other foodborne or diarrhoeal diseases generally associated with poor sanitation and hygiene, thereby providing added value of this program for policy makers and also providing opportunities for integration with water, sanitation and hygiene intervention (WASH) and/or community‐led total sanitation (CLTS) programs in future. The stated willingness of many study respondents to pay for the piloted treatments – particularly PZQ – would also be likely to encourage adoption of the program by local, regional or national governments in future.

While most of the anthropogenic behaviours were well taken up by the study communities, recommendations for confining pigs were less successful. As expressed by one FGD participant, communities and individuals who struggle to provide sufficient food for their own needs cannot afford to divert nutritional resources for confined pigs, even if they appreciate the dangers that scavenging pigs pose for human health. Informal discussions with pig farmers also revealed that corralled pigs were easier targets for potential pig thieves. Similar results and barriers to pig confinement were also reported in other studies (Bardosh, Inthavong, Xayaheuang, & Okello, 2014; Carabin et al., 2018; Lekule & Kyvsgaard, 2003; Thys et al., 2016). Given these restrictions, confining of pigs is not a feasible management strategy in these communities at present, and until sustainable alternative solutions can be found, other control tools must be relied upon to prevent pigs accessing human faeces in these areas. Construction and use of latrines must be encouraged, while also acknowledging the ‘cat method’ of burying faeces when no latrine is available, such as when people are out in the remote planting fields, as a realistic alternative. Some FGD participants proposed that their village headmen could implement by‐laws requiring all households to have latrines; while the Zambian Public Health Act does stipulate that all households must have suitable latrine provision (Zambian Ministry of Health, 2006), enforcement by the local authorities is variable, especially in remote communities. Encouraging involvement of village headmen and/or the area chief in local law enforcement may increase compliance in rural areas.

There was almost unanimous agreement from FGD participants that ASF was responsible for the heavy pig losses in the study areas (from 500 to 700 pigs in each study area in mid‐2015, to approximately 40 pigs in mid‐2017); however, suspicion reportedly remained in some study participants that the CYSTISTOP program activities were causing pig deaths. This appeared to be predominantly associated with hypodermic needle use in pigs, particularly during vaccination and to a lesser extent blood sampling, as opposed to the oral deworming of pigs. It was indeed observed during subsequent intervention rounds that some pig owners refused vaccination for their pigs but allowed the oral treatments, whereas others were reportedly hiding their pigs from the study teams altogether. Many of the FGD participants in the study expressed a desire for an ASF vaccine; if such a vaccine could be developed, and furthermore combined with the anti‐CC TSOL‐18 vaccine, it would no doubt be in great demand throughout the continent.

One key finding from this study has been that the human‐oriented treatments and diagnostic sampling were much better received in these communities than the pig‐oriented interventions. This was not reflected as much in the final intervention data (manuscript in preparation) as in the study teams’ experiences from the field, in which the pig owners, although few in number, required many repeated visits and discussions before some were finally convinced to catch their pigs for interventions. This reluctance may be partly in response to the ASF outbreak as discussed above, with owners potentially considering the few surviving pigs too precious to risk participation in CYSTISTOP study activities, or there may be other underlying sociocultural considerations regarding pig status or management decisions. If a combined CC‐ASF vaccine was developed, it would be likely to greatly enhance pig owner compliance in co‐endemic areas.

The identification of gender‐specific roles and behaviours in this study also provides an opportunity to further tailor educational messages to certain groups to enhance effectiveness. Male‐centric educational messages could focus on the necessity for men to build latrines for their families and could include testimonials of both genders from this and other studies in the district who indicated that a latrine provides dignity and respect for a household, particularly when friends or neighbours come to visit (Thys et al., 2015). The dangers of eating inadequately roasted pork, particularly while drinking alcohol, were also identified by Bardosh et al. (2014) in Lao PDR as a typically male risk factor requiring targeted intervention. The inherent effects of chronic alcohol abuse on susceptibility to *T. solium* infections does not appear to exist in the literature to date, although alcohol consumption is known to be significantly associated with infectious diseases, especially in sub‐Saharan Africa (Rehm, 2011). Furthermore, alcoholic individuals may be more likely to have poor personal hygiene, which is itself a risk factor for CC. For groups of women and children, education could focus on the importance of maintaining handwashing stations with water and soap or ash, ensuring that the faeces of babies and young children are properly disposed of in the latrine, and cooking pork thoroughly to destroy any cysticerci that may be present. Providing similarly targeted leaflets or other educational material including locally broadcast radio programs may further enhance knowledge uptake and retention.

This study has limitations. While attempts were made to minimize bias in the study participants during data collection, individuals may have been reporting socially desirable rather than accurate responses. This may have led to over‐reporting of participation in project activities, exaggerated willingness to pay for treatments and/or under‐reporting of true negative perceptions of the project. Respondents were selected based on recommendations from local community health workers about their suitability, however, they may not have been representative of all individuals, and less common perceptions or responses may not have been captured during data collection. Additionally, the behavioural changes reported by FGD participants were not confirmed by the study team. Follow‐up studies are planned to evaluate the true extent of behavioural change in these communities.

Transmission of the *T. solium* TS/CC complex is known to be driven largely by anthropological behaviours and, like most neglected zoonoses, requires a coordinated multisectoral approach for successful control or elimination (WHO, 2011, 2012). In addition to human and veterinary health, agricultural and water/sanitation sector involvement, this study emphasizes the need for a social and cultural understanding of the biosocial determinants of disease transmission in endemic populations, to identify and address any potential barriers to intervention uptake. Repeated long‐term education sessions are crucial for developing trust of communities, to maximize compliance with interventions and collaboration with longer‐term monitoring/surveillance activities. Engaged and informed communities are also more likely to initiate structural or behavioural changes that prevent disease transmission. In Tanzania, some communities voluntarily implemented local by‐laws regarding latrine usage and maintenance, and restrictions on unnecessary pig transport after receiving *T. solium* education (Lauridsen, 2016).

The success of any control program will be in a large part determined by the acceptance of and support for the proposed interventions by the local communities. Designing long‐term intervention strategies that incorporate biosocial as well as chemotherapeutic and educational strategies is likely to enhance successful adoption of integrated *T. solium* interventions by communities, and thereby increase effectiveness of control or elimination programs in future. This study indicates that *T. solium* control interventions utilizing education and mass drug administration of humans and pigs with oral anthelmintics are generally very well accepted and valued by rural communities in eastern Zambia. While aversion to hypodermic needle use in pigs for vaccination and blood sampling may have sociocultural roots, continued education of and trust building with pig owners may enhance compliance over time. The reported willingness of respondents to pay for the medications would contribute to the feasibility of long‐term, government‐led *T. solium* intervention programs in future. With community support, an integrated program of veterinary, medical and health educational tools can promote substantial behavioural changes and decrease transmission of *T. solium* in rural areas of eastern Zambia.

## CONFLICT OF INTEREST

The study received financial support from the Institute of Tropical Medicine, Antwerp (https://www.itg.be) via the Flemish Department of Science, Economy and Innovation (EWI, https://www.ewi-vlaanderen.be/) and via the Belgian Development Cooperation (DGD, https://diplomatie.belgium.be/en/dgd) in the framework of the collaboration between the Institute of Tropical Medicine in Antwerp, Belgium, and the University of Pretoria, South Africa. Ross University School of Veterinary Medicine provided PhD funding (RUSVM, https://veterinary.rossu.edu/). Praziquantel used in the study was generously donated by the World Health Organisation (WHO, http://www.who.int/). This publication is also based on research funded in part by the Bill & Melinda Gates Foundation and the UK Government through GALVmed (https://www.galvmed.org/; study number ZAF/SUI/15/060). The findings and conclusions contained within are those of the authors and do not necessarily reflect positions or policies of the Bill & Melinda Gates Foundation or the UK Government. The funders had no role in study design, data collection and analysis, decision to publish or preparation of the manuscript. The authors declare no conflict of interests exist.
