# Supplementary material for: Perceptions and acceptability of piloted Taenia solium control and elimination interventions in two endemic communities in eastern Zambia
Source: Transbound Emerg Dis. 2019 Jun 24;67(Suppl 2):69–81. doi: 10.1111/tbed.13214 (PMC7496623; doi:10.1111/tbed.13214)
Supplement: Supplementary file 1 [file TBED-67-69-s001.docx]

**S1 File. Questionnaire used in the e**limination study arm

1. CYSTISTOP is a research project looking to control a disease caused by the pig tapeworm. The project is a collaboration between the University of Zambia and the Institute of Tropical Medicine in Belgium. The research team have already conducted a number of intervention activities with people and with pigs in your village. The aim of this questionnaire is to gain knowledge on the perception of the local communities to the different intervention methods.

2. Do you agree to participate in this questionnaire? (oral consent)

- YES (1)

- NO (2) 🡪 If this response, jump to 45

3. Date of today

4. Name of interviewer

5. Name of village

6. Code of household

7. Surname - Head of household

8. First name - Head of household

9. Name of interviewee

10. Gender of interviewee

- MALE (1)

- FEMALE (2)

11. These questions will be about your perception of the human mass drug administration (MDA) treatments being given as part of the study.

12. Did you (or your children) take the drug?

- YES (1) 🡪 If this response, jump to 14

- NO (2)

13. (If no) Why did you (or your child/ren) not take the drug?

- Not sick (1)

- Scared to take (2)

- Nobody told me to (3)

- No medicine was available (4)

- No permission from husband/grandparent/other (5)

14. Do you know the name of the drug that was given in the MDA?

- Praziquantel (1)

- Niclosamide (2)

- Other (3)

- Don't know (99)

15. Do you know what the drug is used to treat?

- Worms (1)

- Malaria (2)

- Flu (3)

- Schistosomiasis (4)

- Other (5)

- Don't know (99)

16. Did you (or your child/ren) encounter problems when taking the drug?

- YES (1)

- NO (2) 🡪 If this response, jump to 18

17. (If yes) What problems did you encounter? (Tick all that apply)

- Headache (1)

- Dizziness/fainting (2)

- Vomiting (3)

- Diarrhoea (4)

- Joint pain/stiffness (5)

- Rash/hives (6)

- Cough/chest tightness (7)

- Other (8)

18. Will you be happy (for your child/ren) to take the drug at the next intervention visit in 4 months?

- YES (1) 🡪 If this response, jump to 20

- NO (2)

19. (If no) Why not?

- Don't like the side effects (1)

- Need permission (2)

- Won't need the medicine (3)

- Other (4)

20. Would you be willing to pay for the drug for you and your family members?

- YES (1)

- NO (2) 🡪 If this response, jump to 22

21. How much would you be willing to pay? (kwacha for 1 treatment for 1 person)

22. These next questions will be about the pig interventions - oral medication and vaccination - being given as part of the study.

23. Do you know the name of the medication that is being given orally to pigs as part of the study?

- Oxfendazole/Paranthic (1)

- Other (2)

- Don't know (99)

24. Do you know the name of the vaccine that is being given to pigs as part of the study?

- TSOL18 (1)

- S3Pvac (2)

- Other (4)

- Don't know (99)

25. Do you know what disease the medication and vaccine are used to prevent?

- Cysts (1)

- Worms (2)

- African Swine Fever (3)

- Skin rash/lumps (4)

- Diarrhoea (5)

- Paralysis/Convulsions (6)

- Other (7)

- Don't know (99)

26. Do you currently own pigs?

- YES (1)

- NO (2) 🡪 If this response, jump to 37

27. Did you allow your pigs to be treated and vaccinated during this visit?

- YES (1)

- NO (2) 🡪 If this response, jump to 29

28. (If yes) Why did you allow your pigs to be treated and vaccinated?

- To stop the pigs from getting sick (1) 🡪 If this response, jump to 30

- To stop us from getting sick when we eat pork (2) 🡪 If this response, jump to 30

- Everybody is doing it (3) 🡪 If this response, jump to 30

- To get the eartags for the pigs (5) 🡪 If this response, jump to 30

- The veterinary assistant told me to (4) 🡪 If this response, jump to 30

- Other (6) 🡪 If this response, jump to 30

29. (If no) Why did you refuse to allow your pigs to be treated and vaccinated?

- It is not important (1)

- I was not around (2)

- Don't want side effects (3)

- Other (4)

30. Did your pigs have any side effects after the treatments and vaccinations?

- YES (1)

- NO (2) 🡪 If this response, jump to 33

31. (If yes) What were the side effects that your pigs had after the treatment and vaccination? (Tick

all that apply)

- Coughing (1)

- Diarrhoea (2)

- Loss of appetite (3)

- Fever/skin rash (4)

- Vomiting (5)

- Pig/s died (6)

- Other (7)

32. Did you seek assistance for the side effects in your pigs?

- YES- government/private vet clinic (1)

- YES- veterinary assistant (2)

- YES- agricultural officer (3)

- YES- pharmacy/dispensary (4)

- YES- traditional healer (5)

- YES- neighbour (6)

- NO (7)

33. Would you be happy for your pigs to be given the treatment and vaccination at the next intervention visit in 4 months?

- YES (1)

- NO (2)

34. Would you be willing to pay for the treatments and vaccines for your pigs?

- YES (1)

- NO (2) 🡪 If this response, jump to 37

35. (If yes) How much would you be willing to pay for the VACCINE for your pigs? (kwacha per vaccine per pig)

36. (If yes) How much would you be willing to pay for the ORAL MEDICATION for your pigs? (kwacha per treatment per pig)

37. The following questions are about your perception of the human health education being given as part of the study.

38. Did you or your family members take part in any of the health educational activities that were conducted in your village as part of the study?

- YES (1)

- NO (2) 🡪 If this response, jump to 40

39. (if yes) Which of the following health education methods did you or your family members take part in? (Tick all that apply)

- Village sensitisation/education sessions (1) 🡪 If this response, jump to 41

- Read posters displayed at health centre (2) 🡪 If this response, jump to 41

- Read posters delivered to each household (3) 🡪 If this response, jump to 41

- School children attended 'Vicious Worm' workshop (4) 🡪 If this response, jump to 41

40. (If no) Why did you not take part?

- I was working/in field/sick/away (1) 🡪 If this response, jump to 43

- I was looking after a sick household member/baby (2) 🡪 If this response, jump to 43

- No permission from head of household (3) 🡪 If this response, jump to 43

- Not interested (4) 🡪 If this response, jump to 43

- Other (5) 🡪 If this response, jump to 43

41. Have you discussed any of the health education information with others?

- YES- with family members (1)

- YES- with friends (2)

- YES- with neighbours (3)

- NO (4)

42. Which health educational method did you think was best?

- Village sensitisation/education meetings (1)

- Posters at health centre (2)

- Posters given to households (3)

43. Do you know what disease/s the health education is targeting? (Tick all that apply)

- Cysticercosis (*masese/mase/mushokwe*) (1)

- Taeniosis (2)

- Epilepsy (*kunyu*) (3)

- Worms (generally) (4)

- African swine fever (5)

- Other (6)

- Don't know (99)

44. Do you think this disease/s is a serious health problem for people?

- YES (1)

- NO (2)

45. This is the end of the questionnaire. Thank you for your assistance!
