# Supplementary material for: Perceptions and acceptability of piloted Taenia solium control and elimination interventions in two endemic communities in eastern Zambia
Source: Transbound Emerg Dis. 2019 Jun 24;67(Suppl 2):69–81. doi: 10.1111/tbed.13214 (PMC7496623; doi:10.1111/tbed.13214)
Supplement: Supplementary file 2 [file TBED-67-69-s002.docx]

**S2 File. Questionnaire used in the control** study arm

1. CYSTISTOP is a research project looking to control a disease caused by the pig tapeworm. The project is a collaboration between the University of Zambia and the Institute of Tropical Medicine in Belgium. The research team have already conducted a number of intervention activities with people and with pigs in your village. The aim of this questionnaire is to gain knowledge on the perception of the local communities to the different intervention methods.

2. Do you agree to participate in this questionnaire? (oral consent)

- YES (1)

- NO (0) 🡪 If this response, jump to 32

3. Date of today

4. Name of interviewer

5. Name of village

6. Code of household

7. Surname - Head of household

8. First name - Head of household

9. Name of interviewee

10. Gender of interviewee

- MALE (1)

- FEMALE (2)

11. These questions will be about the pig medication being given as part of the study.

12. Do you know the name of the medication that is being given orally to pigs as part of the study?

- Oxfendazole/Paranthic (1)

- Other (2)

- Don't know (99)

13. Do you know what disease the medication is used to prevent?

- Cysts (1)

- Worms (2)

- African swine fever (3)

- Skin rash/lumps (4)

- Diarrhoea (5)

- Paralysis/convulsions (6)

- Other (7)

- Don't know (99)

14. Do you currently own pigs?

- YES (1)

- NO (0) 🡪 If this response, jump to 24

15.Did you allow your pigs to be treated during this visit?

- YES (1)

- NO (0) 🡪 If this response, jump to 17

16. (If yes) Why did you allow your pigs to be treated?

- To stop the pigs from getting sick (1) 🡪 If this response, jump to 18

- To stop us from getting sick when we eat pork (2) 🡪 If this response, jump to 18

- Everybody is doing it (3) 🡪 If this response, jump to 18

- The veterinary assistant told me to (4) 🡪 If this response, jump to 18

- To get the eartags for the pigs (5) 🡪 If this response, jump to 18

- Other (6) 🡪 If this response, jump to 18

17. (If no) Why did you refuse to allow your pigs to be treated?

- It is not important (1) 🡪 If this response, jump to 21

- I was not around (2) 🡪 If this response, jump to 21

- Don't want side effects (3) 🡪 If this response, jump to 21

- Other (4) 🡪 If this response, jump to 21

18. Did your pigs have any side effects after the treatments?

- YES (1)

- NO (0) 🡪 If this response, jump to 21

19. (If yes) What were the side effects that your pigs had after the treatments? (Tick all that apply)

- Coughing (1)

- Diarrhoea (2)

- Loss of appetite (3)

- Fever/skin rash (4)

- Vomiting (5)

- Pig/s died (6)

- Other (7)

20. Did you seek assistance for the side effects in your pigs?

- YES- government/private vet clinic (1)

- YES- veterinary assistant (2)

- YES- agricultural officer (3)

- YES- pharmacy/dispensary (4)

- YES- traditional healer (5)

- YES- neighbour (6)

- NO (0)

21. Would you be happy for your pigs to be given the treatment at the next intervention visit in 12 months?

- YES (1)

- NO (0) 🡪 If this response, jump to 24

22. Would you be willing to pay for the treatments for your pigs?

- YES (1)

- NO (0) 🡪 If this response, jump to 24

23. (If yes) How much would you be willing to pay for the oral treatment for your pigs? (kwacha per treatment per pig)

24. The following questions are about your perception of the human health education being given as part of the study.

25. Did you or your family members take part in any of the health educational activities that were conducted in your village as part of the study?

- YES (1)

- NO (0) 🡪 If this response, jump to 27

26. (If yes) Which of the following health education methods did you or your family members take part in? (Tick all that apply)

- Village sensitisation/education sessions (1) 🡪 If this response, jump to 28

- Read posters displayed at health centre (2) 🡪 If this response, jump to 28

- Read posters delivered to each household (3) 🡪 If this response, jump to 28

- School children attended 'Vicious Worm' workshop (4) 🡪 If this response, jump to 28

27. (If no) Why did you not take part?

- I was working/in field/sick/away (1) 🡪 If this response, jump to 30

- I was looking after a baby/sick household member (2) 🡪 If this response, jump to 30

- No permission from head of household (3) 🡪 If this response, jump to 30

- Not interested (4) 🡪 If this response, jump to 30

- Other (5) 🡪 If this response, jump to 30

28. Have you discussed any of the health education information with others?

- YES- with family members (1)

- YES- with friends (2)

- YES- with neighbours (3)

- NO (0)

29. Which health educational method did you think was best?

- Village sensitisation/education meetings (1)

- Posters at health centre (2)

- Posters given to households (3)

30. Do you know what disease/s the health education is targeting? (Tick all that apply)

- Cysticercosis (*masese/mase/mushokwe*) (1)

- Taeniosis (2)

- Epilepsy (*kunyu*) (3)

- Worms (generally) (4)

- African swine fever (5)

- Other (6)

- Don't know (99)

31. Do you think this disease/s is a serious health problem for people?

- YES (1)

- NO (0)

32. This is the end of the questionnaire. Thank you for your assistance!

for your assistance!
