# Supplementary material for: Perceptions and acceptability of piloted Taenia solium control and elimination interventions in two endemic communities in eastern Zambia
Source: Transbound Emerg Dis. 2019 Jun 24;67(Suppl 2):69–81. doi: 10.1111/tbed.13214 (PMC7496623; doi:10.1111/tbed.13214)
Supplement: Supplementary file 3 [file TBED-67-69-s003.docx]

# S3 File. FGD guides used in elimination and control study arms.

## **FGD guide – Elimination study arm**

Note: these questions refer to all of the interventions that have been conducted in Nyembe since the beginning of the project (in October 2015)

1. Can you tell us about the interventions in your village?
2. What are the advantages/disadvantages for participating in the human MDA?
3. What are the advantages/disadvantages for participating in the pig vaccination and drenching?
   1. Do you see any improvements in the treated pigs?
4. What are the advantages/disadvantages for participating in the health education sessions?
5. What things did you think were done well during the visits?
6. What things did you think could be improved for the next visits?
7. Were you happy with the way the team conducted the intervention visit? Why/not?
8. Any other comments or suggestions?

## **FGD guide – Control study arm**

Note: these questions refer to all of the interventions that have been conducted in Chimvira since the beginning of the project (in October 2015)

1. Can you tell us about the interventions in your village?
2. What are the advantages/disadvantages for participating in the pig vaccination?
   1. Do you see any improvements in the treated pigs?
3. What are the advantages/disadvantages for participating in the health education sessions?
4. What things did you think were done well during the visits?
5. What things did you think could be improved for the next visits?
6. Were you happy with the way the team conducted the intervention visit? Why/not?
7. Any other comments or suggestions?
